# Task planning for sports learning by physical education teachers in the pre-service phase

**DOI:** 10.1371/journal.pone.0212833

**Published:** 2019-03-20

**Authors:** Sebastián Feu, Javier García-Rubio, María de Gracia Gamero, Sergio J. Ibáñez

**Affiliations:** 1 Department of Didactics of Music, Plastic and Body Expression, University of Extremadura, Badajoz, Spain; 2 Faculty of Education, Universidad Autónoma de Chile, Santiago de Chile, Chile; Universidad Nacional de Educacion a Distancia (UNED), SPAIN

## Abstract

Planning the learning task is one of the principal actions that a teacher should engage in, and it is important to know how teachers in the pre-service phase plan learning and communication tasks and the feedback that they use in the classroom. The aim of the present study was twofold: i) to characterize the learning tasks designed by the pre-service physical education teachers; and ii) to identify the relationships between the variables that define the learning tasks and the phases into which a session is structured in Physical Education Teacher Education (PETE) in the pre-service phase. The sample comprised 695 learning tasks designed by fourteen pre-service phase teachers. The independent variable was the lesson structure and the dependent variables were the learning means, the game situation, the game phase, the space where the students practice, the use of the ball in the task, and the kind of feedback provided in the learning tasks. The high predominance of exercises, unspecific games, and no opponent situations, coupled with the low percentage of reflexive feedback, indicates that the pre-service teachers give prevalence to technical over tactical learning. In addition, pre-service teachers show preferences for some of the task characteristics for each part of the lesson structure. Teachers in PETE pre-service phase tasks tend to follow a more traditional methodology, despite having received information about the different methods of sports teaching in their initial training. The current findings seems to indicate a resistance to changing a traditional model for other models centered on game comprehension.

## Introduction

Invasion team sports represent the physical education contents most used in teaching planning [1] and are the most attractive for the students [2]. Invasion team sports are team-based games in which the purpose is to score points while invading the opponent’s territory and keeping the opposing team’s points to a minimum, within a defined time period. Their inclusion in the elementary school curriculum is controversial [3], and has to fulfill some requirements to be considered educational. In this planning, the teachers have to make decisions regarding the learning content and teaching plan, methodology or evaluation, adapting them to the teaching approach that they consider most suitable for achieving the desired learning [4]. One of the basic skills of physical education teachers is planning [5]. This is where the selection and design of learning methods and techniques start. Learning tasks organization is not a product of improvisation or an excess of creativity [6].

There are two main approaches to teaching invasion team sports, the Teacher-Centered Approach and the Student-Centered Approach. Within the Teacher-Centered Approach the Direct Instruction methodology is the most common [7], with the teacher choosing the contents to be developed and managing the class so that the students have more opportunities to respond, get involved cognitively and make decisions about the game [8]. Specifically, the teacher thus designs tasks to develop movement patterns and technical skills that the student has to reproduce. Initially a technical skill is practiced in an unspecific manner in tasks which are isolated from the game to be subsequently incorporated into play [9]. The most commonly used method in direct instruction are unspecific exercises and simple games [10]. The teacher provides the initial information with the criteria for successful performance and a prescriptive feedback to correct errors.

In the Student-Centered Approach, it can be highlighted of Teaching Game for Understanding, TGfU [11]. The TGfU appeared as a practical teaching model for designing tasks that concentrate the students’ attention as they look for solutions that will lead them to understanding the technical aspects of the game. The teacher is responsible for presenting a tactical problem which has to be developed through a series of tasks or games. The TGFU proposal contains 6 stages: play, perception of play, tactical awareness, decision making, technical execution and performance. Worldwide, different proposals have emerged with similar principals, Games Sense [12, 13], Tactical Games, Sport Education Model [14], Play Practice [15], and Concept Based Games [16]. These proposals are based on situated learning, with meaningful and contextualized situations that favor students’ learning [17, 18]. In general, the structure of the tasks is based on forms of play which present a tactical problem in the game that the students have to face with. In order to learning to progress it is important for the teacher to use interrogative feedback to make the students autonomously develop decision making and create their own tactical awareness [7, 19]. Numerous studies confirm that the models based on understanding the game produce improvements over direct instruction with regard to understanding play, decision making, declarative knowledge, enjoyment and motivation in the classroom [20–22].

Learning tasks can be classified according to their degree of specificity, depending on the presence of formal game elements for which they were conceived. Thus they are divided into specific, semi-specific and unspecific tasks [23]. In invasion team sports the design and configuration of the learning tasks are linked to the learning methodologies [24, 25]. Traditional teaching-learning methods, based on direct instruction, prevail in physical education [26]; therefore, the skills are worked on in an isolated from using unspecific or specific global tasks [27].

The tactical games approach uses the most contextualized game situation possible or situated learning [24, 28]. Modifications in the game context facilitate the cognitive connection of the student with the game [18], making it easier to understand its complexity, identify tactical aspects and develop the decision-making process during the game [29]. The game presents a problem that the students must face with using their previous experience and their reflections on practice [30]. Semi-specific or unspecific tasks are also used and in a second phase, specific ones, which present decision-making problems of progressive complexity.

Currently, the analysis of learning tasks is an emerging research line in the field of sports education [31]. The results make it possible to analyze the existing link between the planning of learning tasks and learning methods [27], and knowledge of the pedagogical content [32], or the pedagogical variables [24]. It is important to analyze the learning tasks since these situations provide the practice conditions which allow the players to acquire and execute a sports learning content [31].

Learning tasks could be assessed according to various criteria such as: i) organizational, which serve to improve the practice time of the students, using group organization related aspects, use of space and equipment, and time control; ii) pedagogical, which allow students to understand the content types being worked on, their organization and sequencing [27], such as game phases [10], training means [10], trainee grouping in game situations [24], or the methodology employed by the coaches [32]; and iii) physiological, according to the demands placed on the students, both regarding the internal and external work load [33]. Currently there is a tool available for analyzing learning tasks in sport, the Integral System for Training Task Analysis (SIATE in Spanish) [34], which can be applied in sports and school context. It is a flexible and adjustable tool that can be adapted to diverse invasion team sports and learning contexts. This instrument allows to gather information focused on: *Contextual Data; Coach Data; Session Data; Pedagogical Variables; Organizational Variables; External Work Load Variables; Internal Work Load Variables; and Kinematic Variables* for each of the tasks that comprise a training session.

Pre-service teachers have to be aware of his owns decision consequences’. Tasks’ design plays a major role according the teaching model and, also, the external load imposed in the students. The study and analysis of their practice would help pre-service teacher to develop their teaching skills. Currently few studies exist that analyze the learning tasks designed by teachers for the development of educational goals. There is also a shortage of task design analysis under different methodological approaches, as well as of the learning levels acquired by the students [25]. Analyzed research shows that teachers are not well prepared to plan the training tasks, so PETE students’ analysis of their own practice is important to fill the gap between expert and novices teachers. The knowledge-based reasoning of physical education teachers: A comparison between groups with different expertise. Therefore, the aim of this study was to analyze the tasks planned by teachers in pre-service phase, before their specialization in the physical education area, for the design of a lesson plan on an invasion sport. The general objective was defined in three specific aims: i) To characterize the learning tasks designed by the pre-service physical education teachers from the variables that define a learning task; ii) To identify the relationships among the variables defining the learning tasks and the phases in which a session is structured; and iii) To create a classification on the development of learning tasks in the different phases of the session based on the variables that define them.

## Materials and methods

The study used a comparative transversal associative strategy [35], in which the PETE teachers in the pre-service phase were asked to plan a lesson plan and design tasks to be analyzed from the point of view of the parts of the session.

### Sample

The sample was comprised of 695 units of statistical analysis related to the learning tasks designed by fourteen PETE pre-service teachers. The 695 learning tasks came from the planning done by the pre-service teachers for a specific lesson plan on the development of a sporting content. Basketball was selected as the learning content from among the Invasion team sports.

The fourteen pre-service teachers (female 50%; age: 21.6 ±1.05 yrs), were starting their fourth year of training, and designed their planning before starting their internship stage. The first three years carry out a general education as a teacher, in which they take courses on the different disciplines taught in Primary Education. During these period students only receive a specific course on Physical Education Teaching. In the fourth year, students receive a specific training in physical education. They take four specific courses in Physical Education (24 ECTS credits), an external practicum period as a pre-service teacher (24 ECTS credits), a generalist teacher course (6 ECTS credits) and a final degree project (6 ECTS credits). None of the teachers had qualifications as a coach in any team sport. The lesson plans were developed during 12 lessons with total freedom to include the motor tasks considered appropriate.

### Variables

The independent variable for this research was the lesson structure, which was organized in three stages: warm-up, main activity and culmination activity (this was the last activity before the cool-down). Physical education lessons begin whit the warming up, introducing the aims of the session and setting the student for later effort. In the main part, tasks designed for the development of the objectives of the session are presented. In the cool down activities try to return the body to normal state after vigorous activity.

The dependent variables were chosen to allow definition of the learning tasks. Some of the pedagogical and external load variables defined in the SIATE were selected [34] as follows: *Learning means* (different task type classification); *Game Situation* (number of players involved and the way they were related to the task); *Game Phase* (principal game phase aimed at the sports content worked on in the sports task). The external load variable employed in this study was *Space* (location where the students practiced). Also, two new variables were included: The *presence of a mobile object* (use of the ball in the task); and the *kind of feedback provided* by the teachers.

The study variables were presented to a panel of 11 experts. All the experts fulfilled the criteria of being Sports Science graduates with more than 10 years experience as physical education teachers in Primary School, and articles published in journals the field of didactics. Nine of them had a Ph.D. The experts gave points on a 1 to 10 scale for clarity in the wording of the item (univocity), adequacy regarding the objectives of the assessment (pertinence) and ability to discriminate the information with respect to the study objectives (importance). Aiken’s V was used to evaluate the adequacy of the study variables. Penfield and Giacobbi’s alebraic modified formula was used to calculate the content validity coefficient [36]. The exact critical value, or cutoff point, for accepting Aiken’s V was calculated using the formula proposed by Aiken [37], establishing a value of .83 with a 95% confidence interval. With regard to the pertinenece and importance of all the included variables they were above the critical value of.83 [.84–.93], while regarding the univocity of all the items they were above the critical value for Aiken’s V [.86–93].

### Instrument

The SIATE task analysis instrument [34] was adapted selecting the dimensions and categories that were best suited for analysis in the educational context. Two new specific variables for this study were included in the registration system.

### Proceedings

In Spain, primary school teachers have to pass an educational process at the university with initial training lasting four years. After three years of training as a generalist teacher, the students have a fourth specific year in physical education training, along with an internship period in schools. A group of teachers in the pre-service phase, who were Master in Elementary Education students coursing their fourth year, were asked to design a Lesson Plan with sport as its content. The proposal was to teach an invasion sport that could be played in the school facilities where they were carrying out their teaching practice. Finally, basketball was selected since the resources, materials and facilities were available in every school. The Lesson Plans had to comprise 12 sessions in order to give enough time for learning acquisition, independently of the methodological approach employed in their design [20]. The session parts were also identified and it was recommended to organize in three parts: warm-up, main activity and culmination activity.

The raters who participated in this phase of the study held a Ph.D. and they are experienced in designing and coding teaching and training tasks. The tasks were analyzed using the SIATE instrument and an inter-rater and intra-rater analysis was carried out to guarantee the quality of the data [38]. Following Iguartua (2006), a representative part of the cases was selected as a function of the sample size for the reliability test, which was never smaller than 50 units [39]. Cohen’s Kappa was used to guarantee inter-rater and intra-rater reliability as the variables to be codified were categorical. [40]. It was necessary to use the multi-rater Kappa as there were more than two raters. The raters who participated in this phase of the study were Ph.Ds with ample experience in designing and coding teaching and training tasks. Randolph’s free multi-rater Kappa was used [41] as it is ideal when the raters assign a minimal proportion of agreement to a specific category [42]. The values obtained in the inter-rater reliability test in all the variables that defined the learning tasks were high (*k*_*free*_ >.87), considered as *almost perfect* [43]. In the *game phase* and *learning means* variables the value was slightly lower (*k*_*free*_ >.78) considered as *substantial* concordance [43]. The intra-rater reliability of all grouped variables was *almost perfect* (*k*_*free*_ >.83).

### Statistical analysis

A descriptive exploratory analysis was performed of every variable that defined the learning tasks according to the structure of the physical education lesson. The number of cases and the percentage of each variable are presented in contingency tables.

Secondly, an inferential analysis was used to identify the relations and associations among the study variables. *Pearson’s chi-squared test* (χ2), was used to contrast the hypothesis of independence between the categorical variables analyzed. The association degree between the variable categories was identified with *Cramer’s V coefficient* (φc) [44]. Due to the fact that the Crosstabs Command includes expected frequency distribution lower than 5, and may mask non-significant associations, the Fisher’s exact test was used (Montecarlo adjustment). The association strength was interpreted following the criteria defined by Acock [45]. The *adjusted standardized residuals (ASR)* of the contingency tables were used to interpret the meaning of the associations found in those cases in which the value was greater than |1.96|. Finally, the *correspondence analysis* was employed to illustrate the positive association between variables [46].

A decision tree analysis was used to create the predictive model to illustrate the classification and segmentation of the relation among variables [47]. The CHAID (Chi-squared Automatic Interaction Detector) algorithm [48] was used, since most of the variables were nominal and not binary. This algorithm is one of the most suitable for the social sciences [49]. Exhaustive CHAID method have been used. This method allows to, independently of the categories, analyse with more precision all possible results [50]. A cross validation, with a tree depth of 3 was employed, with a minimum of 75 cases in the filial node and a minimum of 35 cases in the parental node. The statistical software used was the IBM SPSS for Windows version 21 (Armonk, NY: IBM Corp.).

## Results

Table 1 presents the descriptive analysis of the learning tasks planned by the PETE teachers in the pre-service phase for the different parts of the physical education lesson. The most utilized learning mean of the analyzed tasks designed by the teachers was simple exercise (38%) and the unspecific simple game (23.2%). In the warm-up phase simple exercise (45.6%) and unspecific simple game (43.1%) predominate with a scarce presence of more complex games. In the main activity phase they principally used simple exercises (43%) and diverse specific game modalities for the sport: *Modified game* (13.9%), *Specific game* (16.2%) *and Sport* (10.3%).

**Table 1:** Characteristics of the learning tasks in the means and learning situation dimensions.

			Lesson Structure			Total
			Warm-up	Main activity	Culmination activity	
Learning medium	Simple Exercise	*n*	73	167	24	264
		*% Learning medium*	27.7%	63.3%	9.1%	100.0%
		*% Lesson Structure*	45.6%	43.0%	16.3%	38.0%
		*ASR*	2.3	3.1	-6.1	
	Complex Exercise	*n*	1	12	2	15
		*% Learning medium*	6.7%	80.0%	13.3%	100.0%
		*% Lesson Structure*	0.6%	3.1%	1.4%	2.2%
		*ASR*	-1.5	1.9	-.7	
	Unspecific Simple Game	*n*	69	52	40	161
		*% Learning medium*	42.9%	32.3%	24.8%	100.0%
		*% Lesson Structure*	43.1%	13.4%	27.2%	23.2%
		*ASR*	6.8	-6.9	1.3	
	Modified Game	*n*	13	54	31	98
		*% Learning medium*	13.3%	55.1%	31.6%	100.0%
		*% Lesson Structure*	8.1%	13.9%	21.1%	14.1%
		*ASR*	-2.5	-.2	2.7	
	Specific Game	*n*	4	63	16	83
		*% Learning medium*	4.8%	75.9%	19.3%	100.0%
		*% Lesson Structure*	2.5%	16.2%	10.9%	11.9%
		*ASR*	-4.2	3.9	-.4	
	Sport	*n*	0	40	34	74
		*% Learning medium*	0.0%	54.1%	45.9%	100.0%
		*% Lesson Structure*	0.0%	10.3%	23.1%	10.6%
		*ASR*	-5.0	-.3	5.5	
Player Relations	Number Balance 1x1	*n*	64	75	26	165
		*% Regarding players*	38.8%	45.5%	15.8%	100.0%
		*% Lesson Structure*	40.0%	19.3%	17.7%	23.7%
		*ASR*	5.5	-3.1	-1.9	
	Number Balance 2x2	*n*	0	13	5	18
		*% Regarding players*	0.0%	72.2%	27.8%	100.0%
		*% Lesson Structure*	0.0%	3.4%	3.4%	2.6%
		*ASR*	-2.4	1.4	.7	
	Number Balance 3x3 y 4x4	*n*	2	23	15	40
		*% Regarding players*	5.0%	57.5%	37.5%	100.0%
		*% Lesson Structure*	1.2%	5.9%	10.2%	5.8%
		*ASR*	-2.8	.2	2.6	
	Number Balance 5x5	*n*	1	20	7	28
		*% Regarding players*	3.6%	71.4%	25.0%	100.0%
		*% Lesson Structure*	0.6%	5.2%	4.8%	4.0%
		*ASR*	-2.5	1.7	.5	
	Balance nxn	*n*	6	13	28	47
		*% Regarding players*	12.8%	27.7%	59.6%	100.0%
		*% Lesson Structure*	3.8%	3.4%	19.0%	6.8%
		*ASR*	-1.7	-4.0	6.7	
	No opponents 1x0	*n*	55	146	47	248
		*% Regarding players*	22.2%	58.9%	19.0%	100.0%
		*% Lesson Structure*	34.4%	37.6%	32.0%	35.7%
		*ASR*	-.4	1.2	-1.1	
	No opponents 2x0	*n*	28	38	9	75
		*% Regarding players*	37.3%	50.7%	12.0%	100.0%
		*% Lesson Structure*	17.5%	9.8%	6.1%	10.8%
		*ASR*	3.1	-1.0	-2.1	
	No opponents 3x0 and 4x0	*n*	1	16	1	18
		*% Regarding players*	5.6%	88.9%	5.6%	100.0%
		*% Lesson Structure*	0.6%	4.1%	0.7%	2.6%
		*ASR*	-1.8	2.9	-1.6	
	Number imbalance	*n*	3	44	9	56
		*% Regarding players*	5.4%	78.6%	16.1%	100.0%
		*% Lesson Structure*	1.9%	11.3%	6.1%	8.1%
		*ASR*	-3.3	3.6	-1.0	
Game phase	Attack	*n*	100	239	69	408
		*% Game phase*	24.5%	58.6%	16.9%	100.0%
		*% Lesson Structure*	62.5%	61.6%	46.9%	58.7%
		*ASR*	1.1	1.7	-3.3	
	Defense	*n*	15	32	12	59
		*% Game phase*	25.4%	54.2%	20.3%	100.0%
		*% Lesson Structure*	9.4%	8.2%	8.2%	8.5%
		*ASR*	.5	-.3	-.2	
	Mixed	*N*	25	102	53	180
		*% Game phase*	13.9%	56.7%	29.4%	100.0%
		*% Lesson Structure*	15.6%	26.3%	36.1%	25.9%
		*ASR*	-3.4	.3	3.2	
	Other	*N*	20	15	13	48
		*% Game phase*	41.7%	31.2%	27.1%	100.0%
		*% Lesson Structure*	12.5%	3.9%	8.8%	6.9%
		*ASR*	3.2	-3.6	1.0	
	Total	*n*	160	388	147	695
		*% from total*	23.0%	55.8%	21.2%	100.0%

The trainee groups and game situations that were most frequent in the teachers’ planning were 1x0 and 2x0 (46.5%) and 1x1 (23.7%), and it was also observed that individual work prevailed e.g. 1x1 and 1x0 (59.42%). In the warm-up phase 1x1 (40%) and 1x0 (34.4%) predominated; in the main activity 1x0 (37.6%), 1x1 (19.4%) and number inequality (11.3%) were the most common situations. In the culmination phase individual work activities 1x0 (32%) and 1x1 (17.7%) were more common and for the collective game work nxn (19%) and 1x3/4x4 (10.2%).

The results show that most of the tasks were designed to work specifically on the attack phase (*n* = 408), with attack tasks predominating (58.7%) followed by mixed tasks (25.9). The attack content dominated in the three phases of the lesson.

Teachers used activities in reduced spaces, ¼ of the game court (7.2%), very little. In the *warm-up* and *culmination activity* phases full court usage was prioritized, meanwhile in the *main activity* phase half the court was employed. *Ball presence* predominated in the tasks (93.7%), Table 2.

**Table 2:** Characteristics of the learning tasks in space, ball presence and feedback variable dimensions.

			Lesson Structure			Total
			Warm-up	Main activity	Culmination activity	
Space	< ½ game court	*n*	10	30	10	50
		*% Inside the space*	20.0%	60.0%	20.0%	100.0%
		% *Lesson Structure*	6.2%	7.7%	6.8%	7.2%
		*ASR*	-.5	.6	-.2	
	½ game court	*n*	36	203	67	306
		*% Inside the space*	11.8%	66.3%	21.9%	100.0%
		*% Lesson Structure*	22.5%	52.3%	45.6%	44.0%
		*ASR*	-6.3	4.9	.4	
	Full game court	*n*	114	155	70	339
		*% Inside the space*	33.6%	45.7%	20.6%	100.0%
		*% Lesson Structure*	71.2%	39.9%	47.6%	48.8%
		*ASR*	6.5	-5.2	-.3	
Ball presence	No	*n*	16	16	12	44
		*% Ball presence*	36.4%	36.4%	27.3%	100.0%
		*% Lesson Structure*	10.0%	4.1%	8.2%	6.3%
		*ASR*	2.2	-2.7	1.0	
	Yes	*n*	144	372	135	651
		*% Ball presence*	22.1%	57.1%	20.7%	100.0%
		*% Lesson Structure*	90.0%	95.9%	91.8%	93.7%
		*ASR*	-2.2	2.7	-1.0	
Feedback kind	No feedback	*n*	29	2	10	41
		*% Feedback*	70.7%	4.9%	24.4%	100.0%
		*% Lesson Structure*	18.1%	0.5%	6.8%	5.9%
		*ASR*	7.5	-6.8	.5	
	Prescriptive	*n*	54	243	34	331
		*% Feedback*	16.3%	73.4%	10.3%	100.0%
		*% Lesson Structure*	33.8%	62.6%	23.1%	47.6%
		*ASR*	-4.0	8.9	-6.7	
	Interrogative	*n*	13	90	23	126
		*% Feedback*	10.3%	71.4%	18.3%	100.0%
		*% Lesson Structure*	8.1%	23.2%	15.6%	18.1%
		*ASR*	-3.7	3.9	-.9	
	Motivational	*n*	64	53	80	197
		*% Feedback*	32.5%	26.9%	40.6%	100.0%
		*% Lesson Structure*	40.0%	13.7%	54.4%	28.3%
		*ASR*	3.7	-9.7	7.9	

The most commonly used feedback was prescriptive (47.6%) followed by motivational (28.3%) and interrogative (18.1%). In the warm-up (40%) and culmination activity (54.4%) phases, prescriptive feedback was mainly used and prescriptive feedback (62.6%) was the most common in the main activity phase.

**Fig 1:**
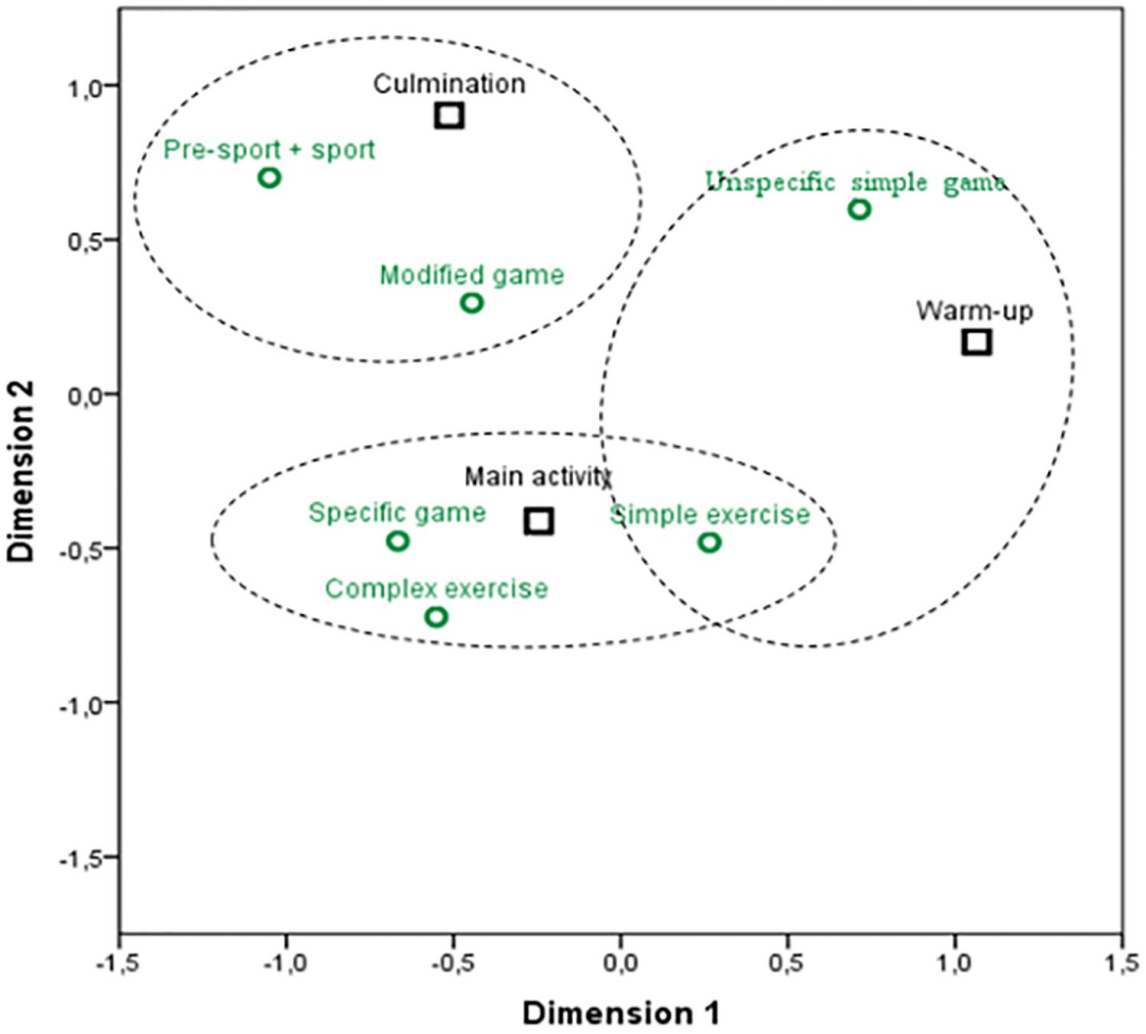
Correlation distribution between the learning means and lesson structure.

An inferential analysis was performed to identify the relation between variables that define the learning tasks and lesson structure. A dependant relation between the learning means and the structure of the lesson (*X*^*2*^ = 137.57; *gl* = 12; *p* < .001/ Fisher’s exact test = 149.85; p < .001) was found. The degree of association between the variable categories was moderate (*φc* = .314; p < .005). In more cases than would be expected, in the warm up part, teachers in the pre-service phase proposed *simple exercises* (*ASR* = 2.3) and *unspecific simple games* (*ASR* = 6.8). On the contrary, in this session phase, *modified games* (*ASR* = -2.5), *specific games* (*ASR* = -4.2) and *pre-sport-sport* (*ASR* = -4.6) were employed on fewer occasions than expected. In the main activity phase of the lesson there were more learning tasks than expected of *simple exercises* (*ASR* = 3.1) and *specific games* (*ASR* = 3.9). However, there were less cases than expected of *unspecific simple games* (*ASR* = -6.9). Finally, in the culmination activity part, the learning means were diverse. The results show that there were more cases than expected using *pre-sport or sport* (*ASR* = 5.5) and *modified games* (*ASR* = 2.7) and less cases than expected of *simple exercises* (*ASR* = -6.1). The correlation analysis (Fig 1) shows the positive significant associations identified in the contingency tables.

**Fig 2:**
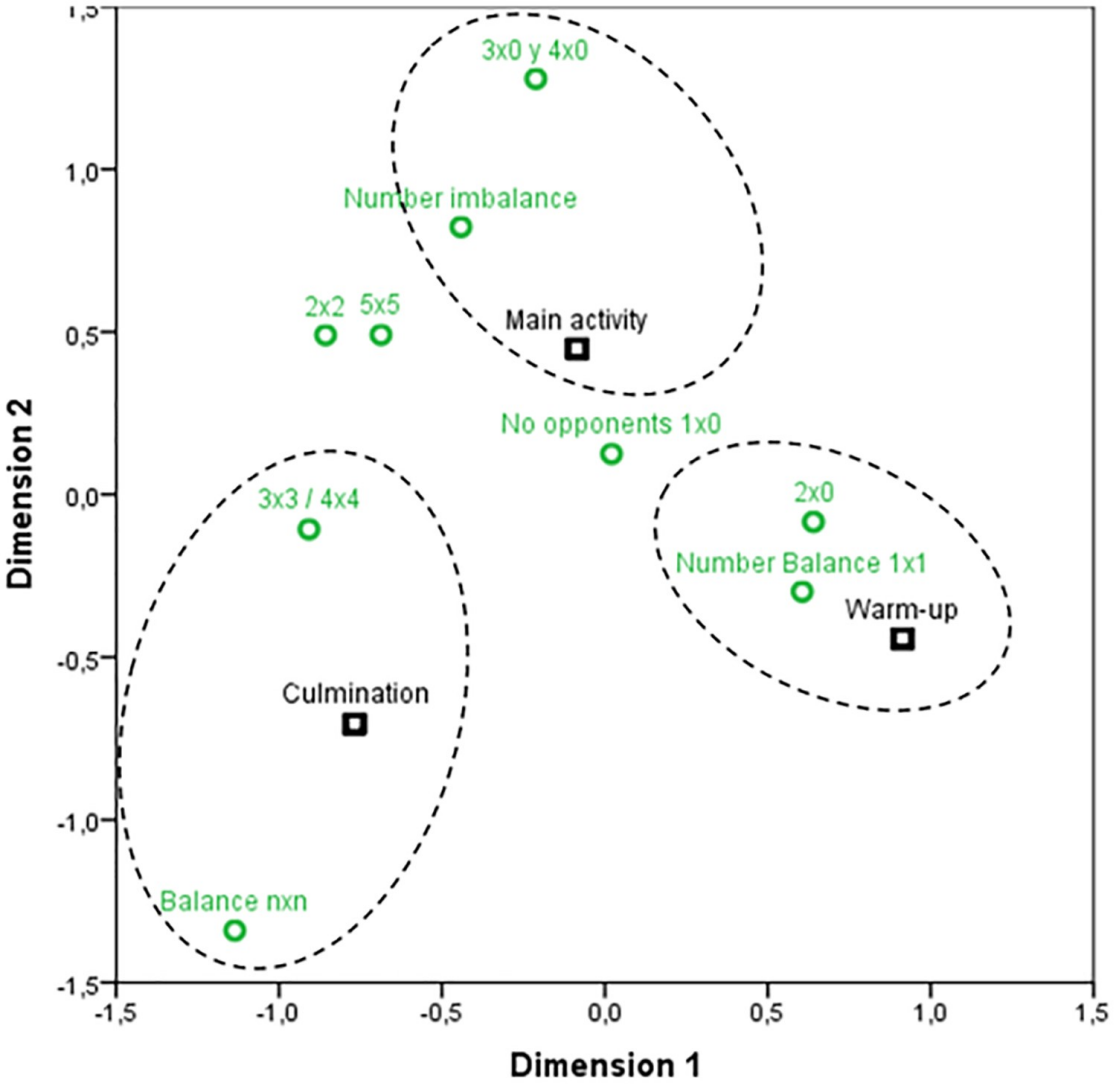
Correlation distribution between the game situations and session structure.

The chi-square statistic shows that there is a dependent relation between the game situations and session parts (*X*^*2*^ = 119.52; *gl* = 16; *p* < .001/ Fisher’s exact test = 111.44; p < .001), with a moderate association (*φc* = .293; *p* < .005). The contingency table analysis and the correlation chart (Fig 2) indicate that in the warm up phase there were more cases than expected of 1x1 (*ASR* = 5.5) and 2x0 (*ASR* = 3.1) situations. On the other hand, there were less 2x2, 3x3, 4x4, 5x5 and number imbalance (*ASR* = -2.4 to -3.3) situations. In the main activity phase the prevalence of the individual work situations is noteworthy, 56.96% with 1x0 (n = 146) and 1x1 (n = 75) tasks. In the main activity phase there were more cases of 3x3/4x4 and number imbalance situations (*ASR* = 2.9 and 3.6 respectively) than expected and less cases than expected of 1x1 and nxn situations (*ASR* = -3.1 and -4.0, respectively). Lastly, in the culmination activity phase, the results show that there were more cases of 3x3 and 4x4 (*ASR* = 2.6) and nxn situations (*ASR* = 6.7) than expected.

**Fig 3:**
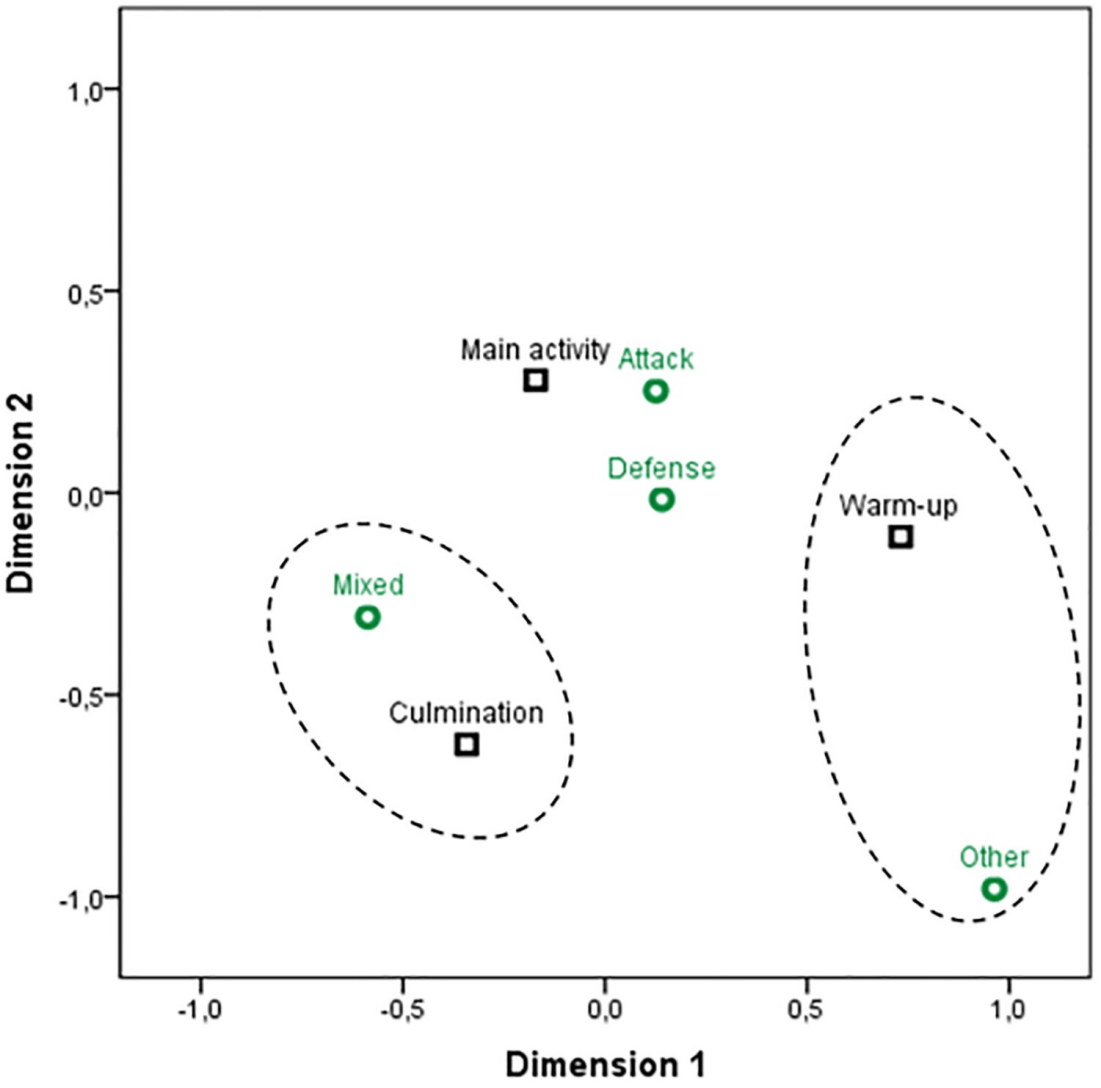
Correlation distribution between the game phases and the session structure.

Significant associations were found between the *Game phases* (*X*^*2*^ = 30.24; *gl* = 6; *p* < .001/ Fisher’s exact test = 30.52; p < .001) and *Session parts* variables with a low strength (*φc* = .148; *p* < .001). In the correlation chart, Fig 3, it can be seen that in the warm-up phase there were more cases than expected of tasks that are not developed in any phase of the game (*ASR* = 3.2) and less cases than expected of working with mixed objectives, meaning attack and defense (*ASR* = -3.4). In the main activity phase there were less cases than expected of tasks without specific goals in the game (*ASR* = -3.6). In the culmination activity phase attack objectives were more prevalent, although there were fewer cases than expected compared to the other tasks (*ASR* = -3.3), however there were more tasks with mixed objectives (*ASR* = 3.2).

*Space* usage (*X*^*2*^ = 46.17; *gl* = 4; *p* < .001 / Fisher’s exact test = 47.87; p < .001) was associated with the lesson phases, although this association was weak (*C* = .250; *p* < .001). In the warm-up phase full court activities predominated (*ASR* = 6.5), while in the main activity phase half court usage prevailed (*ASR* = 4.9) and there were fewer cases than expected of full court activities (*ASR* = -5.2). Tasks in a reduced space, less than half the court, were scarce, representing just 7.2%.

*Ball presence* was dominant in the tasks (93.7%). A significant association (*X*^*2*^ = 7.65; *gl* = 2; *p* < .05 / Fisher’s exact test = 129.81; p < .001) was found between ball presence in tasks and lesson parts where they were used, although this association was low (*φc* = .105; *p* < .05).

**Fig 4:**
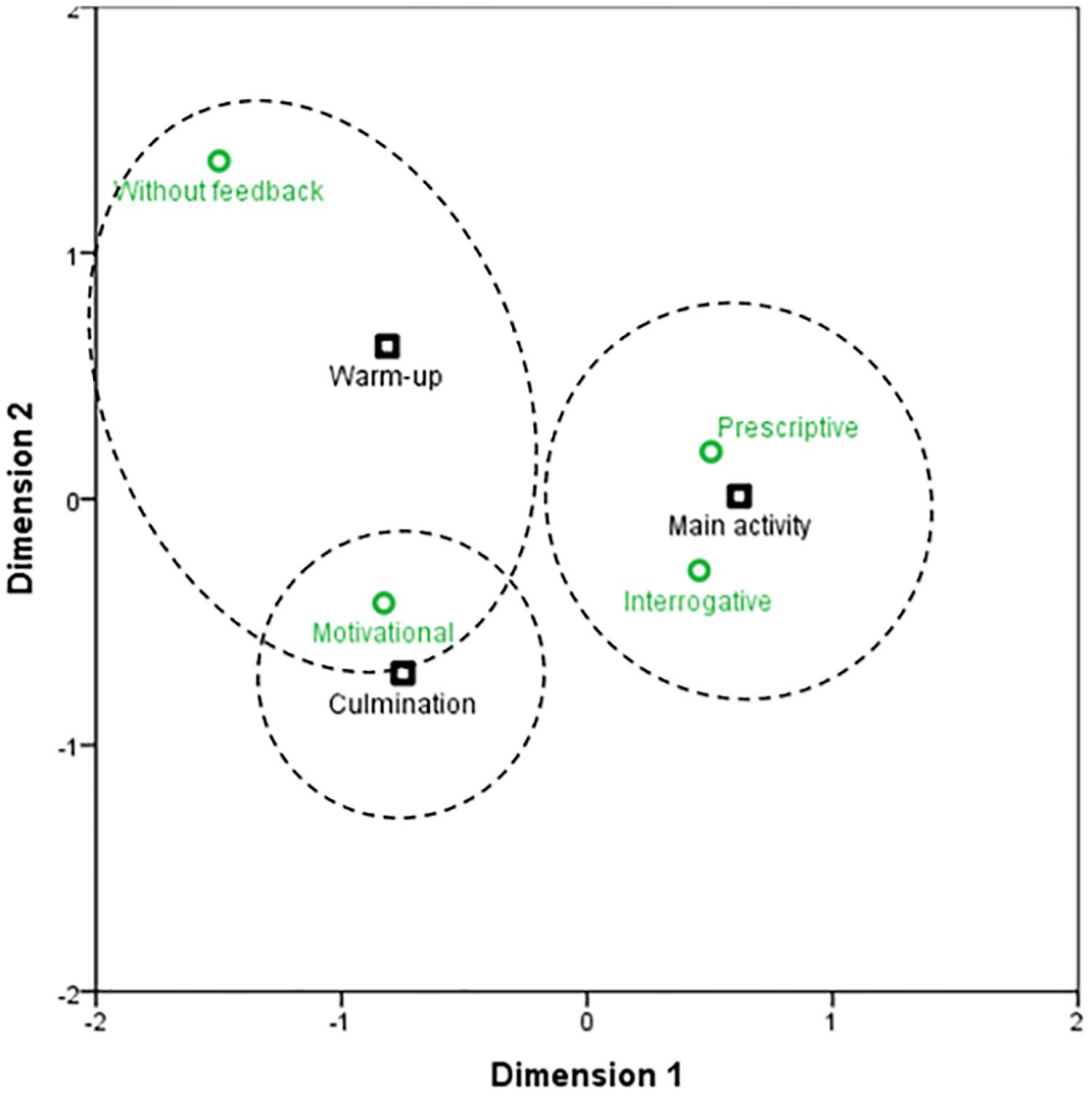
Correlation distribution between the planned feedback and session structure.

Lastly, the analysis showed significant associations between *planned feedback* in the task (*X*^*2*^ = 190.41; *gl* = 6; *p* < .001/ Fisher’s exact test = 190.50; p < .001) and the parts of the lesson with a moderate association (*φc* = .370; *p* < .005). The correlation chart (Fig 4) shows that in the warm-up phase there were more cases of motivational feedback (*ASR* = 3.7) and less cases than expected of prescriptive feedback (*ASR* = -4.0); also, it is noticeable that in this phase there were contents without planned feedback (*ASR* = 7.5). The future teachers employed the prescriptive (*ASR* = 8.9) and interrogative feedback (*ASR* = 3.9) more in the main activity of the lesson, showing fewer cases than expected of motivational feedback (*ASR* = 9.7).

The decision tree technique was used to predict what kind of tasks the teachers employed in the three required parts of the lesson. Learning means, Game phase, Player relation, Space, Mobile object presence and Kind of feedback were included in the model. The exhaustive CHAID algorithm showed a risk of an estimated .345 in the cross validation, with an error of .018. In general, 67.3% of the tasks were correctly classified, although specifically the warm-up (37.5%) and culmination activity tasks (51%) were classified much lower than the main activity phase tasks (85.8%).

**Fig 5:**
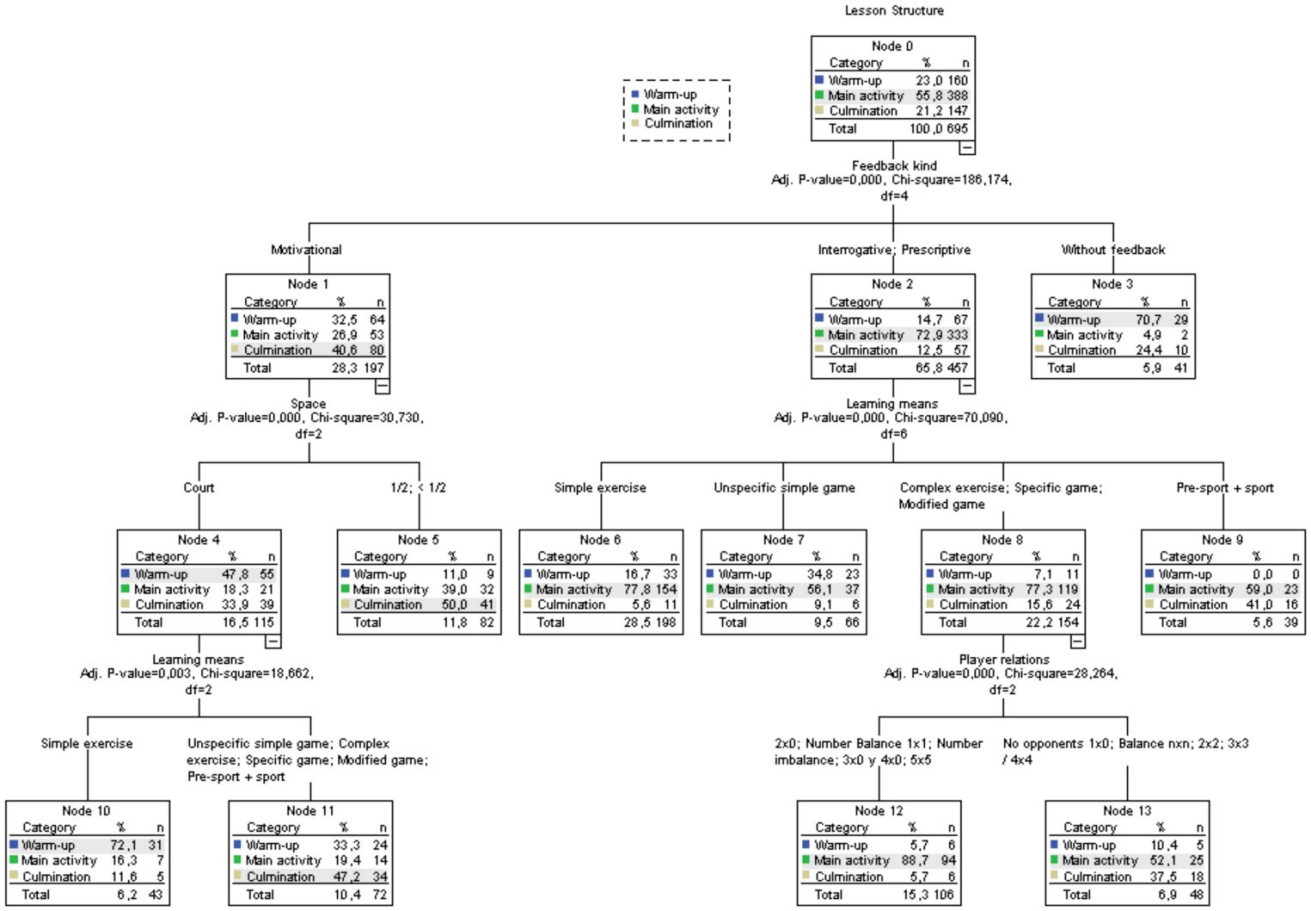
Decision tree using the exhaustive CHAID algorithm to predict the task characteristics in the session parts.

The decision tree comprises fourteen nodes. In the zero node the highest percentage of tasks was developed in the main activity phase (55.8%), Fig 5. The dependant variable branches into three nodes belonging to the task feedback variable, with node 2, interrogative and prescriptive feedback, showing a higher chi-squared value (*X*^*2*^ = 70.090; *df* = 6; *p* < .001). Node 2 re-branches into four lines, nodes 6 to 9, with node 6, Simple exercise, grouping more tasks (*n* = 198), followed by node 8 Complex exercise, Specific Game and Modified game (*n* = 154). Node 8 re-branches (*X*^*2*^ = 28.264; *df* = 6; *p* < .001), with the variable relation between the players in nodes 12 and 13. Node 12 comprises activities without opponents (2x0, 3x0, 4x0), individual game 1x1 and collective game 5x5 situations, meanwhile node 13 is formed by collective game situations and small sided games (2x2, 3x3, 4x4) and individual situations (1x0).

Node 1, motivational feedback (*X*^*2*^ = 30.730; *df* = 6; *p* < .001), gives rise to nodes 4 and 5, belonging to the space variable, with node 4 re-branching in the Learning means variable (*X*^*2*^ = 18.662; *df* = 6; *p* < .001), nodes 10 and 11. Node 4 shows that fullest court tasks are developed in the warm-up phase (47.85%), meanwhile the use of the half court in tasks with motivational feedback occurred in the culmination activity phase (50%). Node 10 shows that in full court activities with motivational feedback the most commonly used tasks in the warm-up phase were the Simple exercises (72.1) meanwhile in node 11 in the culmination activity phase there was a diverse amount of learning means with greater difficulty (Fig 5).

## Discussion

The general objective of this study was to analyze the tasks planned by teachers in the pre-service phase before their specialization in the physical education area. During their internships in schools, these teachers plan the lesson plans of an invasion sport such as basketball using mainly simple exercises followed by unspecific simple game as the learning-teaching means. Only one third of the means are contextualized in sports game problems, employing modified games, the specific game and the sport. Moreover, half the game situations have no opponents, with the 1x0 situation predominating. The most utilized situation from those without opponents is the 1x1. Finally, the most worked on game phase is the attack phase.

An analysis of the employed learning means (exercises and simple games) and learning situations (mainly without opponents) seems to indicate that teachers in the pre-service phase do not employ tasks that are specific and contextualized in the sport itself, which would imply that they are more inclined towards traditional teaching, from the point of view of the methods used [27]. The motor responses of the students are initially defined in the tasks, with a limited margin for decision making [51]. Similar results have been found in Secondary Education/High School where the teachers employ a high volume of decontextualized and clearly defined tasks [52]. Some authors consider that these closed activities are associated with inexperienced teachers, since they apparently give them greater control of the situation. Also the use of models centered on understanding the game provides less security for inexperienced teachers. Likewise, some experience is necessary in order to manipulate and modify the game situations through restrictions and rules [53] that allow the teacher to create new game problems. All of this causes a lack of confidence in the teachers in the designing of tasks using this approach [54]. These results differ from the ones found in coaches of school-age children in the extra-curricular context, where 1x1 situations followed by 2x2 situations prevailed [24].

Furthermore, the PETE teachers in the pre-service phase mainly designed attack phase contents. This tendency matches the results obtained from analyzing basketball teaching for these ages in after-school sport [10]. Beginning in the attack phase increases the students’ motivation, with the development of attack and defense contents evolving in an undulating manner [55].

It is noticeable that half the feedback planned by the teachers is of the descriptive and/or prescriptive type, with motivational feedback also being used. Similar results were found in the teaching of collective sports in the Mandatory Secondary Education/High School, where the feedback was mainly prescriptive and affective, followed by reflexive [52]. Interrogatory feedback is the least employed. Teachers do not plan topics ahead to foster reflection among the students. The PETE teacher knows that different types of feedback allow different objectives to be achieved, as is demonstrated when they vary their use during the lesson. They start with motivational feedback, evolving during the main activity phase to mostly prescriptive. The comprehensive methodology is based on the stimulation of the students’ reflection to adapt their knowledge to the conditions of practice [18]. The designing of quality questions should be an integral part of the teacher’s planning process [56], and this is one of the greatest difficulties experienced by teachers for applying a model centered on game comprehension [54].

One of the principles of game comprehension centered models is the modification of learning situations through the exaggeration and simplification of the elements of the sports game. In the PETE teachers’ planning, half the learning means are exercises and simple games, employing no opponent situations, with interrogative feedback being the least utilized choice. These characteristics show that the teaching process follows a traditional approach [57].

In the PE lesson, the PETE teachers in the pre-service phase show a preference for some of the task characteristics. In the warm-up phase they employ mostly simple exercises and unspecific simple games, eliminating warm-ups with analytical activities, such as exercises, and replacing them with unspecific games [58]. They use fewer learning means related to the game, such as modified games, specific game, pre-sport and sport. Also, the most employed game situations in this phase are 1x1 and activities without an opponent, 1x0 and 2x0. From a cognitive point of view, these activities performed in the warm-up phase are less difficult because of the lack of rules and the fact that they are oriented towards physical activation and learning technical abilities.

In the main activity phase the use of simple exercises prevails, while in the last phase of the lesson, played situations predominate. Although in this part of the lesson there is an increase in the means based on the game, in general, the teaching methodology is more centered on the traditional model [59]. Furthermore, in the main activity there are fewer cases than expected of 1x1 activities and the activities without opponent are maintained, greatly increasing the group situations without defense (3x0; 4x0) and with unbalanced numbers. Basic situations without opponents (1x0, 2x0, … 5x0) and easy commands favors the teachers’ control sensation. Also, the easy reproduction by students of these types of routines generated an efficacy thinking on pre-service teachers. This perception allow teachers to prefer teacher centered methods. In addition, previous experiences in teacher centered approach in sport learning or the scarce availability of specific literature about lessons design in students centered approach could lead to avoid these students centered methods [60].

The PETE teachers in the pre-service phase were also required to design an activity as the culmination event of the session. In this phase, the most employed method was the simple game. Moreover, it was observed that the specific tasks increase, as a learned content application method during the session, with more modified games and sport; balanced number games significantly increase, 3x3, 4x4 and nxn, coming closer to being centered on game comprehension [59].

Game content selection, for attacking and defending phases, is an important part of the lesson plan. The PETE teachers in the pre-service phase designed tasks with different contents as a function of the game phase and part of the session. Attack game phase contents predominate in the work of these PETE teachers. The warm-up phase contains tasks without an orientation towards game content. Possibly these activities are oriented towards physiological warm-up and the development of basic motor skills. In the culmination activity phase there is a significant increase in mixed (attack and defense) tasks, probably because these tasks have been designed for the application of knowledge in the real game. In general the PETE teachers employed more attack tasks, probably due to the motivation attached to attack [61]. On the other hand, without previous attack content work it is very difficult to develop defensive actions [62]. Tasks using a ball are mostly used, using the full court for the warm-up phase and the half court and small sided game, for the main activity.

Feedback is part of the teacher-student communication process being one of the actions that allow orientation of the task. In the warm-up phase motivational feedback or no feedback activities prevail, with no clear orientation towards any teaching-learning method. In the main activity phase of the session, the feedback is mostly prescriptive, and occasionally interrogative, a significant increase compared to the previous phase. In the culmination activity phase the motivational feedback greatly increases. Interrogative or reflexive feedback is not employed for the students to verbalize key ideas for task resolution.

In summary, the high predominance of exercises, unspecific games, and no opponent situations, together with the low percentage of reflexive feedback, indicates that the teaching gives prevalence to technical over tactical learning [32], based on the practice of decontextualized and isolated tasks before their application to the real game [29], representing a traditional teaching-learning model [52]. This decomposition and elimination of the game elements in the task design leads to a learning process isolated from the real sport practice scenarios [63]. Contextualized, or situated, learning, with modifications of the game situations, allows the application of students’ previous knowledge favoring their understanding of the game [18], and their motivation. These tasks are designed with the modification of rules and specific game elements, such as partner players, adversaries, mobile objects and game space, and can be aimed towards the development of contents in the attack and defense phases.

In general, the observed characteristics of the tasks designed by the PETE teachers in the pre-service phase are closer to a traditional methodology, despite their having received information about the different methods of sports teaching in their initial training. This seems to indicate a resistance to changing a traditional model for other models centered on game comprehension. It is more than likely that the decisions made by these teachers are based on implicit ideas and not on empirical-scientific or academic evidence [64]. Therefore, it is necessary to explore the teachers’ previous beliefs and knowledge since they influence their choice and the development of the teaching profession. It is very important for pre-service teachers to have real experiences in PE lessons during their teaching. This experience can have a direct impact on their behaviors and decisions when planning. Pre-service teachers need to experience students centered approaches during their formation. Reflexive thinking about these models, previous experiences as school students and athletes, and their present practice in real context could lead to avoid to repeat teacher centered approach.

### Practical applications

Teacher training centers have to emphasize a paradigm shift in the model of the future teachers. As was observed in this work, theoretical-practical academic training is not enough to orientate the teachers towards more constructive models. The study plans have to be concerned with provoking meaningful experiences for the students and foster reflective processes in order to contrast previous experiences with the learning models centered on game comprehension, considering the advantages for school learning and how to overcome the difficulties of their application in the classroom. Likewise, the practical phase of the PETE teachers has to be reconsidered; encouraging reflection to overcome their implicit beliefs and favoring the transmission of practical knowledge according to the constructive based learning models through mentoring.
